# Molecular profiling of human non-small cell lung cancer by single-cell RNA-seq

**DOI:** 10.1186/s13073-022-01089-9

**Published:** 2022-08-13

**Authors:** Qingqing Li, Rui Wang, Zhenlin Yang, Wen Li, Jingwei Yang, Zhijie Wang, Hua Bai, Yueli Cui, Yanhua Tian, Zixin Wu, Yuqing Guo, Jiachen Xu, Lu Wen, Jie He, Fuchou Tang, Jie Wang

**Affiliations:** 1 Biomedical Pioneering Innovation Center, School of Life Sciences, Peking University, Beijing, China; 2 State Key Laboratory of Molecular Oncology, National Cancer Center/National Clinical Research Center for Cancer/Cancer Hospital, Chinese Academy of Medical Sciences & Peking Union Medical College, Beijing, China; 3 Beijing Advanced Innovation Center for Genomics & Ministry of Education Key Laboratory of Cell Proliferation and Differentiation, Beijing, China; 4 Academy for Advanced Interdisciplinary Studies, Peking University, Beijing, China; 5 Peking-Tsinghua Center for Life Sciences, Peking University, Beijing, China

**Keywords:** Single-cell RNA sequencing, Non-small cell lung cancer, Mixed-lineage cancer cells, Tumor heterogeneity

## Abstract

**Background:**

Lung cancer, one of the most common malignant tumors, exhibits high inter- and intra-tumor heterogeneity which contributes significantly to treatment resistance and failure. Single-cell RNA sequencing (scRNA-seq) has been widely used to dissect the cellular composition and characterize the molecular properties of cancer cells and their tumor microenvironment in lung cancer. However, the transcriptomic heterogeneity among various cancer cells in non-small cell lung cancer (NSCLC) warrants further illustration.

**Methods:**

To comprehensively analyze the molecular heterogeneity of NSCLC, we performed high-precision single-cell RNA-seq analyses on 7364 individual cells from tumor tissues and matched normal tissues from 19 primary lung cancer patients and 1 pulmonary chondroid hamartoma patient.

**Results:**

In 6 of 16 patients sequenced, we identified a significant proportion of cancer cells simultaneously expressing classical marker genes for two or even three histologic subtypes of NSCLC—adenocarcinoma (ADC), squamous cell carcinoma (SCC), and neuroendocrine tumor (NET) in the same individual cell, which we defined as mixed-lineage tumor cells; this was verified by both co-immunostaining and RNA in situ hybridization. These data suggest that mixed-lineage tumor cells are highly plastic with mixed features of different types of NSCLC. Both copy number variation (CNV) patterns and mitochondrial mutations clearly showed that the mixed-lineage and single-lineage tumor cells from the same patient had common tumor ancestors rather than different origins. Moreover, we revealed that patients with high mixed-lineage features of different cancer subtypes had worse survival than patients with low mixed-lineage features, indicating that mixed-lineage tumor features were associated with poorer prognosis. In addition, gene signatures specific to mixed-lineage tumor cells were identified, including *AKR1B1*. Gene knockdown and small molecule inhibition of *AKR1B1* can significantly decrease cell proliferation and promote cell apoptosis, suggesting that *AKR1B1* plays an important role in tumorigenesis and can serve as a candidate target for tumor therapy of NSCLC patients with mixed-lineage tumor features.

**Conclusions:**

In summary, our work provides novel insights into the tumor heterogeneity of NSCLC in terms of the identification of prevalent mixed-lineage subpopulations of cancer cells with combined signatures of SCC, ADC, and NET and offers clues for potential treatment strategies in these patients.

**Supplementary Information:**

The online version contains supplementary material available at 10.1186/s13073-022-01089-9.

## Background

Lung cancer is one of the most common malignant tumors with the highest incidence and morbidity according to the GLOBCAN 2018 [1]. There are two major histological subtypes of lung cancer: small cell lung cancer (SCLC) and non-small cell lung cancer (NSCLC) [2]. NSCLC, accounting for 84% of lung cancer cases, mainly includes three histological subtypes: adenocarcinoma (ADC), squamous cell carcinoma (SCC), and large cell carcinoma (LCC). Accumulating evidence suggests that NSCLC has high heterogeneity, not only with different histologies but also with different molecular and cellular features [3, 4].

Accurately identifying the subtypes of lung cancer is important for its clinical treatment. In clinical practice, lung cancer subtypes are mainly identified through a combination of histological features and immunological markers. In general, ADCs have glandular histology or mucin production and express hallmark genes such as TTF1 (also known as NKX2-1), NAPSA, cytokeratin 7 (KRT7), and MUC1 [5]. SCCs have squamous differentiation features and are clinically diagnosed by immunostaining of p63 or p40 (TP63), cytokeratin 5/6 (KRT5/KRT6), and transcription factor SRY-box 2 (SOX2) [6]. Finally, SCLCs and large cell neuroendocrine carcinoma (LCNEC) are neuroendocrine tumors (NETs) that usually have neuroendocrine differentiation properties and are defined by the specific expression of chromogranin A (CHGA), synaptophysin (SYP), and neural cell adhesion molecule 1 (NCAM1) [7].

The potentially distinct cell origins of the lung cancer subtypes also contribute to the heterogeneity of lung cancers at tumor initiation. The exact cell origins of lung cancer subtypes are still elusive, but previous studies revealed that SCCs, SCLCs, and ADCs are roughly distributed in the lung following a proximal-to-distal pattern; furthermore, these three major subtypes originate from different lineages of cells in the lung [8]. Genetically engineered mouse models showed that AT2 cells in the alveoli are the predominant cell-of-origin of ADCs, although club cells can also give rise to ADCs [9, 10]. SCCs are generally considered to arise from basal cells in proximal airways, and SCLCs are speculated to originate from pulmonary NE cells [11–14]. Although the three lung cancer subtypes originated from different lineages, recent works reported that the tumor state transitions can occur with cellular program dysregulations in epigenetics and transcriptomics during tumor development [15–17].

As the lung is a complex organ composed of more than 40 different types of cells [18, 19], including epithelial, endothelial, stromal, and immune cells, it is important to separate different cell types to profile their characteristics. Due to technological limitations, conventional sequencing methods require millions of cells. Hence, studies on NSCLC were limited to bulk samples, and these results only showed the average molecular features of NSCLC, concealing the intratumoral heterogeneity. With the development of high-throughput sequencing techniques, especially single-cell sequencing techniques, detailed molecular characterization of lung cancer is now feasible. Recent advances in single-cell RNA sequencing (scRNA-seq) studies on NSCLC provided a comprehensive cellular diversity landscape within lung tumor tissues. Previous studies characterized the stromal cells such as T cells, macrophages, fibroblasts, and endothelial cells in the lung tumor microenvironment [20–23], dissected the tumor heterogeneity and cellular reprogramming in advanced and metastasis lung cancer [24–26], comparatively analyzed the differences in heterogeneity and cellular compositions between ADC and SCC [27], and revealed the molecular mechanisms of tumor therapy-induced tumor evolution and resistance [28]. However, efforts in profiling lung tumor cells of primary NSCLC and understanding their cellular biology by scRNA-seq are still limited. In this study, we performed high-precision single-cell RNA-seq analysis with surgical tissues from 19 primary lung cancer patients as well as 1 pulmonary chondroid hamartoma patient. By analyzing the epithelial cells from tumor tissues and matched normal tissues of 16 patients at the single-cell transcriptome level, we identified an interesting subpopulation of mixed-lineage cancer cells simultaneously expressing classical marker genes for two or even three histologic subtypes of NSCLC. Then, by in-depth analyzing the characteristics of mixed-lineage cancer cells, we identified gene signatures specific to mixed-lineage cancer cells, including AKR1B1. Functional analysis verified that AKR1B1 was necessary for tumor growth, suggesting that it can serve as a candidate target for tumor therapy of NSCLC patients with mixed-lineage features. Our work provides novel insights into the molecular characteristics of NSCLC.

## Methods

### Clinical human specimen collection

In this study, we collected 19 primary lung cancer patients (14 ADC patients, 3 SCC patients, 1 combined small cell lung cancer (C-SCLC) patient, and 1 patient with mixed adenocarcinoma and neuroendocrine carcinoma (MANEC)) and 1 pulmonary chondroid hamartoma patient undergoing surgical resections. All patients were diagnosed based on histologic diagnosis and tumor cellularity by pathologists. Patient clinical information such as age, gender, TNM classification, and stage can be found in Additional file 1: Fig. S2a. The cancer subtypes were identified according to the 2015 WHO classification. All patients signed an informed consent prior to enrollment and tissue donation. Fresh tissues were stored on ice in RPMI-1640 medium supplemented with 10% fetal bovine serum (FBS) and 1% penicillin/streptomycin for dissociation. Cells from all patients were used for single-cell RNA-seq analysis. Cells from some of the patients were also used for bulk whole-genome sequencing. This study was approved by the Research and Ethics Committee of the Chinese Academy of Medical Sciences & Peking Union Medical College (NCC1798).

### Cell lines and culturing

Human H2009 and SW480 cell lines were obtained from the Cell Resource Center, Peking Union Medical College (which is the headquarter of the National Infrastructure of Cell Line Resource, NSTI) and cultured in DMEM/F12 supplemented with 10% fetal bovine serum (FBS) and 1% penicillin/streptomycin antibiotic cocktail. H2009 cell line served in this study was verified by short tandem repeat STR typing. Both cell lines were validated to be free of mycoplasma contamination.

### Histopathology

Histopathology images were obtained from Cancer Hospital, Chinese Academy of Medical Sciences , where 3-μm-thick sections were prepared for staining with hematoxylin and eosin for further examination.

### Dissociation of single cells from lung tissues

Freshly resected lung tissues were dissected in RPMI-1640 medium + 10% FBS on ice and washed with ice-cold Dulbecco’s phosphate-buffered saline (DPBS; Corning). Then, the tissue was transferred to a tube and was cut into small pieces with a scissor. Next, the tissue was resuspended in 1 ml of digestion buffer consisting of Collagenase Type I (2 mg/ml; Gibco), Dispase II (1 mg/ml; Millipore), and DNase I (0.2 mg/ml; Roche) in RPMI-1640 medium and incubated at 37 °C for 30–40 min with frequent agitation. The tissue was gently pipetted up and down 40–50 times, and the suspension was filtered through 100-μm mesh filters. After being centrifuged at 500 × *g* for 10 min at 4 °C, the cell pellet was re-suspended in red blood cell lysis buffer and incubated at RT for 3 min. Then, the cells were washed again, and the cell pellet was re-suspended with RPMI-1640 medium +10% FBS. To guarantee the cells for single-cell RNA-seq with high cell viability, we checked the cells for viability via trypan blue staining after tissue dissociation for each sample we collected. If the cell viability is lower than 80%, the samples would be given up and will not be processed for single-cell transcriptome sequencing.

### Single-cell RNA-seq library construction and sequencing

Single-cell cDNA amplification was performed based on a modified single-cell tagged reverse-transcription sequencing (STRT-seq) protocol [29, 30]. Briefly, a single clean cell with integral morphology and high cell viability under the sight of × 100 magnification microscope was randomly picked into the lysis buffer by mouth pipette. Then, the released mRNAs were reverse transcribed into cDNA by poly(T) primer anchored with cell-specific barcodes and 8-bp unique molecular identifiers (UMIs). Followed by second-strand cDNA synthesis and pre-amplification, the amplified cDNAs can be pooled together, fragmented, and the 3′ ends were used for library construction using Kapa Hyper Prep Kit (Kapa Biosystems). The constructed libraries were sequenced on HiSeq 4000 system as paired-end 150-bp reads.

### Bulk DNA extraction and library construction

Genomic DNA of tumor tissues and matched normal tissues were extracted using the DNeasy Blood & Tissue Kit (QIAGEN) according to the manufacturer’s specification. The DNA concentrations were quantified using a Qubit Fluorometer and Qubit dsDNA HS Assay Kit (Invitrogen), and the qualities were evaluated with Fragment Analyzer (AATI). Approximately 500 ng genomic DNA was sheared by Covaris S2, subsequently for library construction with KAPA Hyper Prep Kit. The samples were sequenced on HiSeq 2500 system as paired-end 150-bp reads.

### Bulk RNA extraction and library construction

H2009 cells were seeded on 6-cm cell culture dishes and were transfected with two different siRNAs targeting *AKR1B1* and a non-targeting siRNA control (NC) using lipofectamine RNAIMAX (Thermo Life). After transfected for 48 h, cells were harvested for bulk RNA sequencing. Briefly, about 5 × 10^5^ cells were used for total RNA extraction by the RNeasy Mini Kit (Qiagen) according to the manufacturer’s instructions. Total RNA quality was assessed by Nanodrop ND-100 (Wilmington). The mRNA was isolated using the NEBNext Poly (A) mRNA Magnetic Isolation Module (New England Biolabs). The RNA library was constructed using the NEBNext ultra RNA library prep kit for Illumina (New England Biolabs). The constructed libraries were sequenced on Illumina HiSeq 4000 system to generate paired-end 150-bp reads.

### Multiplex fluorescent immunohistochemistry staining

Collected fresh lung tissue resections were fixed in 4% paraformaldehyde, followed by dehydration and embedding in paraffin as routine protocol. Subsequently, the paraffin blocks were cut into 3-μm-thick sections and adhered to the slices for Multiplex fluorescent IHC staining. Briefly, the sections were firstly placed in a 65 °C oven for 1 h and deparaffinized in xylene, followed by rehydrated successively in 95%, 80%, and 70% alcohol. Then, antigen retrieval was performed by incubating in critic acid buffer at 95 °C for 20 min. Endogenous peroxidase was blocked by incubating in 3% H_2_O_2_ at room temperature (RT) for 10 min. Subsequently, 10% normal goat serum was added at RT for 1 h to block non-specific sites. Then, the sections were incubated with freshly diluted primary antibodies at RT for 1h. The primary antibodies that were used for cancer type validation included anti-p63 (Abcam, clone EPR5701, diluted at 1:12000), anti-TTF1 (Abcam, clone EP1584Y, diluted at 1:250), and anti-PGP9.5 (UCHL1) (Abcam, clone EPR4118, diluted at 1:250). Next, the second antibody was added to the sections and incubated at RT for another 1 h. Finally, the sections were incubated in freshly prepared fluorochrome at RT for 10 min. The antigen binding sites were visualized and analyzed with the PerkinElmer Vectra Automated Multispectral Imaging System.

### RNA in situ hybridization

Fresh lung tissues were washed with ice-cold DPBS and fixed in fresh cold 4% paraformaldehyde overnight at 4 °C. Then, the tissues were washed with DPBS for three times and subsequently were dehydrated by incubating in 30% sucrose until the tissues sank. The fixed tissues were embedded in Tissue-Tek O.C.T. Compound (#4583, Sakura) and were frozen at − 80 °C for cryostat sections; 12-μm-thick sections were prepared for RNA in situ hybridization. RNA in situ hybridization was performed with the RNAscope Multiplex Fluorescent Reagent kit (Advanced Cell Diagnostics) according to the manufacturer’s protocol. Opal 520 and Opal 570 fluorophores were used at 1:1500 dilution.

### Cell proliferation assays

To assess the effects of *AKR1B1* on cell proliferation, H2009 cells were transfected with two different siRNAs targeting *AKR1B1* and NC in parallel using lipofectamine RNAIMAX (Thermo Life). After transfection for 24 h, cells were reseeded into 96-well plates at 3 × 10^3^ cells/well. Cells were incubated with CellTiter-Glo® Reagent for 5 min on a shaker to induce cell lysis and were incubated for another 10 min at room temperature. Then, the cell numbers were detected based on the quantitation of ATP using a luminometer. Cell proliferation was monitored every day. Each experiment was conducted in triple technical replicates. Sequences of siRNAs were as follows: siAKR1B1-1: 5′GUGAAAGCUAUUGGCAUCUTT3′; siAKR1B1-2: 5′CCAGUACUGCCAGUCCAAATT3′; and siRNA for NC: 5′ UUCUCCGAACGUGUCACGUTT3′.

### Cell apoptosis assays

Cell apoptosis assays were performed using Annexin V-FITC/PI (bb-4101, BestBio) according to the manufacturer’s instructions. Briefly, H2009 and SW480 cell lines were treated with epalrestat or DMSO for 48 h, respectively, and then, the cells were harvested and centrifuged at 2000 rpm for 5 min, resuspended in 300-μl binding buffer containing 5 μl Annexin V-FITC, and incubated in the room temperature for 15 min. Subsequently, 5 μl PI was added to the cells to incubate another 5 min at RT before they were analyzed by flow cytometry. Experiments were performed in triple technical replicates.

### Animal experiments

Animal experiments were conducted following the standard procedures approved by the committee on the Ethics of Animal Experiments of the Health Science Center of Peking University. Mice were housed in ventilated cages with up to four per cage in an animal barrier facility at Peking University. All cages were sterilely changed weekly and supplied with hardwood bedding. All mice were maintained in a specific pathogen-free room at 22 to 26 °C with a 12-h shift of light-dark schedule and fed with sterile pellet food and autoclaved water provided ad libitum. Approximately 5 × 10^5^ H2009 cells were suspended in 50 μl PBS and were mixed with equal volumes of Matrigel, and then, the suspension was subcutaneously injected into the flanks of 6-week-old female NOD-SCID mice. Subsequently, the mice were administered intragastrically with 75 mg/kg/day epalrestat or the same volume of sterile water every day. Animals were monitored regularly. Once signs of morbidity were observed or the subcutaneous tumor size was required for sacrifice, the mice would be euthanized. Tumor volume was calculated with a caplier: tumor volume = 1/2 (length × width^2^). After treatment for 36 days, the mice were euthanized by carbon dioxide inhalation, and the tumors were collected to evaluate the tumor volume and weight.

### Quality control and mapping of single-cell transcriptome data

Firstly, paired-end sequencing data were demultiplexed according to the 8 bp cell barcode in the Read-2, and then, the 8-bp UMI sequence in Read-2 was attached to the corresponding Read-1. Then, low quality, poly A, TSO, and adapter contaminated reads were removed by our quality-control pipeline. Then, the clean reads were mapped to the human genome (hg19) by TopHat (v.2.0.14) [31], and the uniquely mapped reads were kept for further analysis. HTSeq were used to calculate the abundance of transcripts with unique UMIs for each gene [32]. Finally, the gene expression levels were normalized into transcripts per million reads (TPM).

### Copy number variantions (CNVs) inferred with single-cell RNA-seq data

We used previously published methods to deduce the CNVs of every single cell [33, 34]. In brief, the CNV score for each gene was calculated as the average expression level of 100 genes around the gene on the chromosome. All normal epithelial cells were used as control, and the CNV scores of tumor cells were centered to zero by subtracting the average CNV score of all normal epithelial cells. Next, for the 10M window, the relative CNV score was calculated by averaging the CNV scores of all genes within the window. Finally, the CNV patterns of tumor cells were visualized and clustered with the “pheatmap” package, and the CNV clusters with cell numbers more than 10 were kept in the heatmap.

### The confirmation of CNVs with bulk whole-genome sequencing data

Firstly, low-quality and adapter contaminated reads were removed with our quality control pipeline. Then, clean reads were aligned to the human genome (hg19) with BWA (version 0.7.5a). Next, reads were assigned to the 10M window, and the reads of each window were normalized by the total reads of each sample. Finally, the CNV patterns were plotted as a dot plot.

### Mitochondria mutation tracing with single-cell RNA-seq data

The mapped bam files produced by Tophat were used for mitochondrial mutation calling following the GATK suggested pipeline for RNA-seq data (https://github.com/gatk-workflows/gatk4-mitochondria-pipeline) [35]. To accelerate the calling process, we only called mutations on the mitochondria. The detailed code can be found on GitHub (https://github.com/WRui/) (https://github.com/wrui/pdf).

### Dimensionality reduction and clustering

**Fig. 1:**
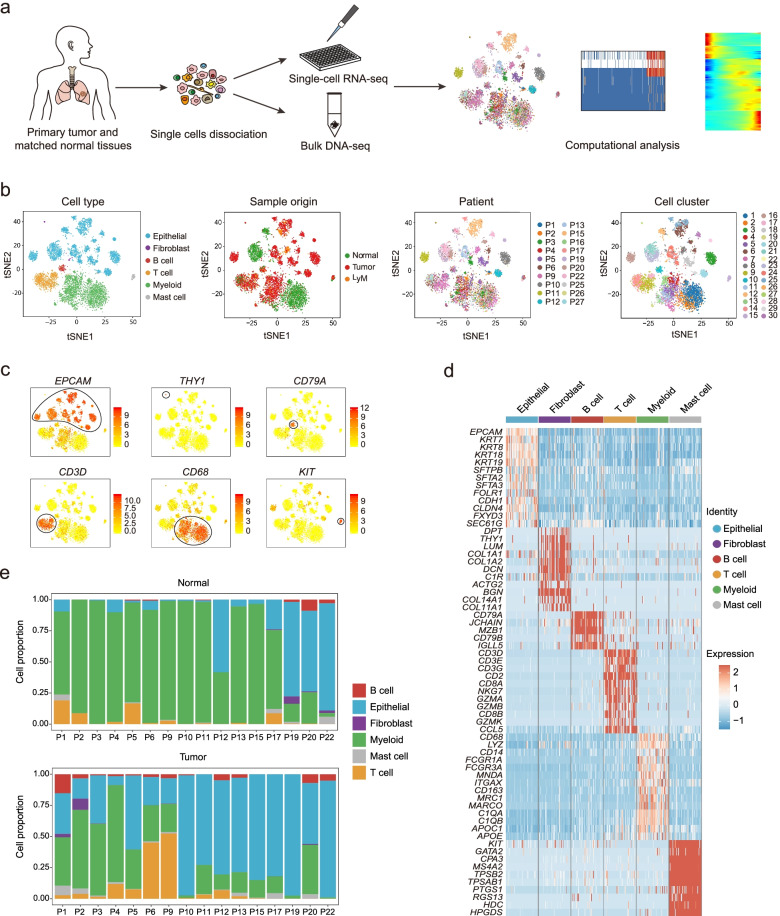
Single-cell transcriptome atlas of primary lung cancer. **a** Schematic diagram showing the experimental workflow of this study. Primary lung tumor tissues and matched normal tissues were collected from 19 primary lung cancer patients as well as 1 pulmonary chondroid patient who underwent surgery. After we got the freshly resected tumor tissues and matched normal tissues, we dissociated the tissues into single-cell suspension and rapidly picked the single cell into cell lysis buffer for single-cell RNA-seq analysis. **b** t-SNE plot of 7364 high-quality single cells showing the cell type identification, sample regions (LyM represents lymph node metastasis), patient information, and cell cluster information. **c** Expression levels of canonical cell type markers across 7364 single cells. **d** Heatmap showing the expression of specific cell type markers in six major cell types. **e** Proportions of identified six major cell types in normal tissues and tumor tissues separately across patients

The SCENIC package was used to establish a gene regulatory network and to cluster our single-cell RNA-seq data of 7364 cells with inferred gene regulatory network simultaneously (Fig. 1b) [36]. Following the SCENIC manual, the python version was applied to our data with the default setting. Cells were clustered into 30 clusters and were annotated as epithelial cell, T cell, B cell, myeloid, fibroblast, and mast cell with well-known cell type marker genes (Fig. 1b). After the cell clusters were determined, cells were visualized with tSNE in the Seurat package (v3.0.2) [37] (Fig. 1b).

### Identification of differentially expressed genes

The “FindAllMarkers” function in the Seurat package (v3.0.2) [37] was used to identify differentially expressed genes (DEGs) among several groups with parameters (logfc.threshold = 1.5, min.pct = 0.25, only.pos = T).

### Pseudotime analysis of non-small cell lung cancer

To construct the single-cell trajectories along the NSCLC tumorigenesis, the DEGs between all normal and tumor epithelial cells were firstly identified with the Seurat package (v3.0.2). Then, these genes were used to reconstruct the trajectories with the Monocle2 software [38]. The function “reduceDimension” in monocle2 was used to reduce the dimensionality with parameters “max_components = 2,” method = “DDRTree.” Next, cells were ordered into a low-dimensional space with “orderCells” function.

### Cancer type definition and visualization

**Fig. 2:**
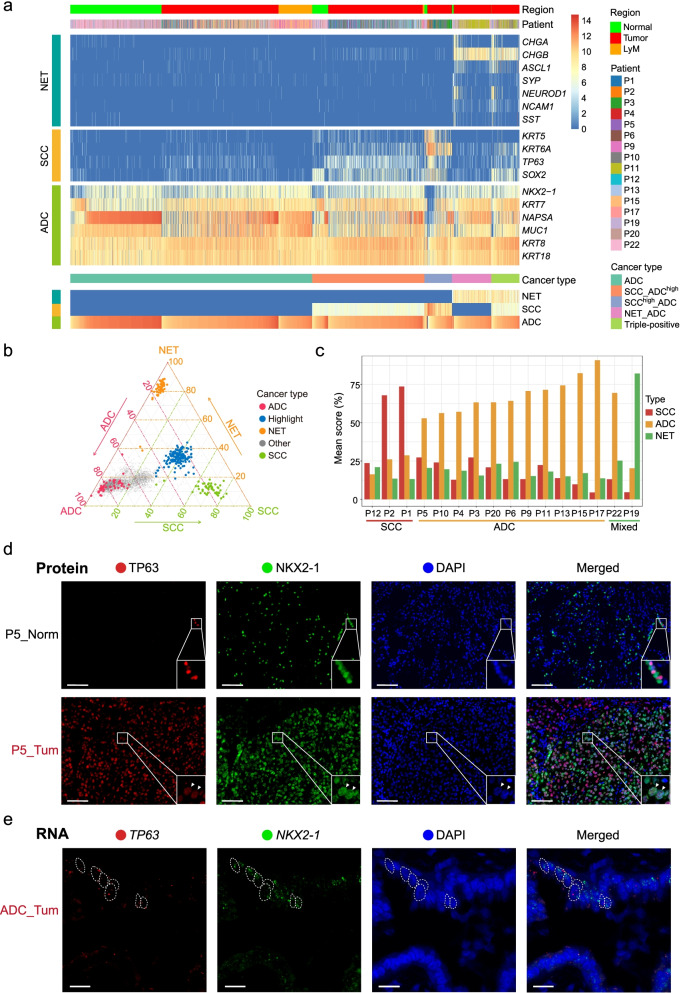
Different lineage markers are co-expressed in the same individual tumor cells. **a** Heatmap showing the unsupervised hierarchical clustering of 3373 normal and tumor epithelial cells from 16 patients with the expression levels of NET, SCC, and ADC classical lineage markers. **b** Lineage score analysis further confirmed our cancer type identification. The ggtern plot displayed the cancer type score for each individual cell based on the expression levels of these cancer subtype lineage markers of SCC, ADC, and NET. “Highlight” cells represented cells from patient P12 with higher mixed features of ADC, SCC, and NEC. “Other” cells referred to the remaining cells except for ADC cells, NET cells, SCC cells, and “highlight” cells. **c** Barplot showing the component score for SCC, ADC, and NET cancer subtypes across every patient. **d** Multiplex fluorescent IHC staining of cells from patient P5 with p63 (TP63, SCC marker) and TTF1 (NKX2-1, ADC marker). Arrows indicated the double-positive cancer cells. Scale bar, 100 μm. **e** RNA in situ hybridization for *TP63* and *NKX2-1* in primary ADC tissues. Scale bar, 100 μm

To define cancer type of cells, we clustered cells by expression of cancer type associated genes: CHGA, CHGB, ASCL1, SYP, NEUROD1, NCAM1, and SST for neuroendocrine carcinoma; KRT5, KRT6A, TP63, and SOX2 for squamous cell carcinoma; and NKX2-1, KRT7, NAPSA, MUC1, KRT8, and KRT18 for adenocarcinoma (related to Fig. 2a). For each cell, we firstly filter out these cancer type-associated genes whose expression level (log2(TPM+1)) is lower than 5. Then, the highest expression levels of genes associated with each cancer type were defined as the corresponding cancer type attribution score. Then, through clustering the cancer-type attribution of each cell, we grouped cells into five groups. According to the cancer type gene expression pattern of each group, we finally redefined the cancer subtype: ADC, SCC_ADC^high^, SCC^high^_ADC, NET_ADC, and triple-positive.

The following several marker genes are commonly used to distinguish between adenocarcinoma (*NKX2-1*, *KRT7*, *NAPSA*), squamous cell carcinoma (*TP63*, *KRT6A*, *KRT5*), and neuroendocrine carcinoma (*CHGB*, *NCAM1*, *SYP*). To find DEGs among different cancer types, we firstly calculated the mean expression levels of these three cancer type marker genes in each individual tumor cell, and for each cancer cell type, the top 50 cells that highly expressed corresponding cancer type markers were selected for DEG identification among adenocarcinoma, squamous cell carcinoma, and neuroendocrine carcinoma. Next, we used these newly identified top 50 DEGs for each cancer type to score other tumor cells by calculating the total expression of specific cancer type genes. Next, for each cancer type, we scale the score to range from 0 to 100. Then, the scores for these three cancer types were visualized with the “ggtern” R package (Related to Fig. 2b; Additional file 1: Fig. S3c).

### Cancer cell type identification from published datasets

To validate the existence of mixed-lineage cancer cells, 1710 alveolar cells, 214 epithelial cells, and 7447 cancer cells characterized by Lambrechts et al. [20] and 4356 human non-immune cells (excluding endothelial cells, fibroblasts, and smooth muscle cells) characterized by Zilionis et al. [22] were used for cancer type identification. The expression levels of lineage-specific markers **(***NKX2-1*, *KRT7*, and *NAPSA* for ADC; *TP63*, *KRT5*, and *KRT6A* for SCC; *CHGB*, *SYP*, and *NCAM1* for NET) were used for cancer type score calculation. Only cells expressing any lineage markers were kept for further exploration. Cancer types were defined using the same analysis method as mentioned above.

### TCGA lung ADC and SCC data download and survival analysis

The TCGA mRNA expression data and patient clinical information were downloaded through the R package “cgdsr.” Then, we used the cancer type genes we identified from our single-cell dataset to score the bulk TCGA dataset. First, we calculated the total expression of these cancer type genes for each sample and then divided the tumor score by the sum of the three tumor scores (A, S, N). A represents adenocarcinoma (ADC), S represents squamous cell carcinoma (SCC), and N represents neuroendocrine tumors (NET). Finally, we used the score to define the lineage-mixed features of tumor:

$$\mathrm{score}\;=\;\mathrm c\;(1-\max\;(\mathrm A,\;\mathrm S,\;\mathrm N))/\max\;(\mathrm A,\;\mathrm S,\;\mathrm N)$$

In this way, for each sample, the higher the score is, the higher the mixed degree of the samples. Then, we ranked the samples and separated them into low scores and high scores. For ADC samples, we divided the samples based on the score = 1. For SCC samples, we divided the samples based on the score = 1.33. Then, we used the R package “survival” to investigate the correlation between the lineage-mixing score and patient survival.

### Bulk RNA-seq analysis

The raw sequencing reads were firstly trimmed to remove low-quality and adapter-contaminated reads. Then, the clean reads were mapped to the human genome (hg19) with TopHat, and the gene expression levels were calculated with HTSeq and then normalized to TPM. The R package “DESeq2” [39] was used to identify DEGs between siAKR1B1 samples and NC samples. For DEGs, *p*-value < 10^−15^ were used to draw the heatmap. Gene Ontology analysis of DEGs was conducted using Metascape (http://metascape.org).

## Results

### Transcriptomic landscape and cell type classification of NSCLC

To analyze the transcriptional characteristics of NSCLC, we performed high-precision scRNA-seq analysis with tissues from 19 primary lung cancer patients who underwent surgery, including 14 ADC patients, 3 SCC patients, 1 combined small cell lung cancer (C-SCLC) patient, and 1 patient with mixed adenocarcinoma and neuroendocrine carcinoma (MANEC) [40]. Cells from healthy normal tissues from a pulmonary chondroid hamartoma (PCH) patient were collected as a control (Fig. 1a; Additional file 1: Figs. S1, S2a). In total, we obtained the single-cell transcriptome of 9002 cells. After stringent filtering (Additional file 1: Fig. S2b), we retained 7364 (81.8%) high-quality individual cells for subsequent analyses (Additional file 1: Fig. S2a).

To classify major cell types, we performed a *t*-distributed stochastic neighbor embedding (t-SNE) analysis using SCENIC identified cell clusters [36] (Fig. 1b). Based on the expression patterns of known canonical cell type markers, we identified six major cell types, including epithelial cells (*EPCAM*), fibroblasts (*THY1*), B cells (*CD79A*), T cells (*CD3D*), myeloid cells (*CD68*), and mast cells (*KIT*) (Fig. 1c, d). We observed variations in the proportions of these six cell types in the 16 patients sequenced without cell preselection (Fig. 1e). To comprehensively analyze the molecular characteristics of cancer cells, we focused our study on epithelial cells. Finally, we totally obtained 3373 epithelial cells from 16 patients of normal and tumor tissues for subsequent analysis, and the cell distribution in each patient was shown in Additional file 1: Fig. S2c.

### scRNA-seq uncovered mixed-lineage tumor cells in NSCLC

In this study, three different lung cancer subtypes were covered: ADC, SCC, and NET. To further distinguish these lung cancer lineages at the single-cell level, we classified the cancer subtypes based on the expression of clinically well-established markers (Fig. 2a). According to the gene expression patterns of cancer subtype-specific markers, we redefined cancer types for each tumor epithelial cell, namely, ADC, SCC_ADC^high^, SCC^high^_ADC, NET_ADC, and triple-positive (coexpressing ADC/SCC/NET markers) (Fig. 2a, the details are in the “Methods” section). Intriguingly, we found that 55–98% of cancer cells simultaneously expressed classical marker genes (*NKX2-1*, *KRT7*, and *NAPSA* for ADC; *TP63*, *KRT5*, and *KRT6A* for SCC; *CHGB*, *SYP*, and *NCAM1* for NET) for two or even three different histologic subtypes of NSCLC in the same individual cell (defined as a mixed-lineage cell) in six patients (P5, P10, P11, P13, P19, and P22). If we extend the marker genes to include the non-classical ones such as *MUC1* for ADC, *SOX2* for SCC, and *ASCL1* for NET, the mixed-lineage cancer cells were notably observed in all of the sixteen patients from whom we isolated epithelial cells in tumor tissues. The ratio of the redefined cancer subtype across every patient is demonstrated in Additional file 1: Fig. S3a. To the best of our knowledge, this is the first study to identify mixed-lineage cancer cells at the whole-transcriptome level.

Next, to investigate the molecular heterogeneity of mixed-lineage cancer cells, we performed principal component analysis (PCA) for 1400 mixed-lineage cancer cells (Additional file 1: Fig. S3b) and found that they were not separated by cancer subtypes; instead, different cancer subtypes were mixed together within the same patients, which indicating that heterogeneity across patient was greater than cancer types within the patient. Furthermore, for these three lineages of cancer cells, we selected the top 50 individual cells that showed the strongest lineage-specific marker gene expression signatures for each lineage and performed PCA (Additional file 1: Fig. S3c). The cells of these three different lineages were divided into three independent clusters accordingly, which indicated that these 150 selected cancer cells accurately represent the unique molecular features of the ADC, SCC, and NET lineages. Therefore, we performed differentially expressed gene (DEG) analysis for these cancer cells to identify new markers for each subtype (Additional file 1: Fig. S3d; Additional file 2: Table S1). We found that the cancer subtype markers (*GRP*, *CHGB*, *NEUROD1*, and *CHGA* for NET; *KRT5*, *DSC3*, *KRT6B*, *TP63*, and *KRT6A* for SCC; and *NKX2-1*, *KRT7*, *NAPSA*, and *MUC1* for ADC) were specifically expressed in the corresponding cancer subtypes. Furthermore, genes such as *TUBB3* and *MEST* for NET, *TRIM29* and *CSTA* for SCC, and *CEACAM6* for ADC can be used as candidate markers to distinguish these three subtypes.

To verify the identity of each individual cell, we entered all tumor epithelial cells into a ggtern plot with cells scored based on the expression levels of markers specific for the ADC, SCC, and NET lineages (Fig. 2b). Consistent with the above cell clustering and cancer type redefinition data (Fig. 2a), the ggtern plot also showed that some intermediate cells lay in the middle among the ADC, SCC, and NET lineages, indicating their mixed-lineage nature (Fig. 2b). Specifically, the majority of cells from patient P12 displayed the molecular features of all three lineages (Additional file 1: Fig. S3e). Next, the principal component scores of these three lineages for each patient were counted (Fig. 2c). Among the three patients diagnosed with SCCs, P12 showed a lower SCC score than P1 and P2. Meanwhile, ADC patients P5 and P10 displayed lower ADC scores than the other patients with ADCs. For patient P22 with MANEC, the ADC score was higher than the NET score, indicating that P22 had stronger ADC characteristics. All the results above showed that there were mixed-lineage cancer cells that coexpressed marker genes of different cancer lineages in NSCLC at the whole-transcriptome level. Moreover, we analyzed the expression of the lineage-specific marker genes from previously published datasets using the same method [20, 22]. 12.4% (237 out of 1910) and 11.8% (432 out of 3668) of the cells in the tumor tissue were identified as mixed-lineage tumor cells, respectively (Additional file 1: Fig. S3f). In the former dataset downloaded, the information about every single cell is from which individual patient was missing, and we cannot trace the origin of the mixed-lineage tumor cells to each individual patient. However, in the latter dataset, mixed-lineage cancer cells were identified in every one of the seven patients analyzed, with the ratio ranging from 7.3 to 24.8%. These results clearly confirmed the prevalent existence of mixed-lineage tumor cells in NSCLC patients.

NKX2-1 and TP63 are the best-known markers to discriminate between ADC and SCC. In our study, we found that four ADC patients (P5, P10, P11, and P13) had a large proportion of tumor cells coexpressing *NKX2-1* and *TP63* (Additional file 1: Fig. S4a). To further confirm this, we performed multiplex fluorescent immunohistochemistry (IHC) staining. For P5, the tumor areas on the section were strongly and diffusely positive for NKX2-1 and TP63, and 51% (35,654 out of 70,292 cells analyzed) of the individual cancer cells co-expressed these two markers simultaneously (Fig. 2d). In contrast, cells from adjacent normal tissues did not show double positivity for NKX2-1 and TP63. Therefore, multiplex fluorescent IHC staining verified our scRNA-seq results at the protein level. For patients P10 and P11, the staining results also showed the presence of NKX2-1 and TP63 double-positive cells in tumor tissues (Additional file 1: Fig. S4b). Surprisingly, we found that many cancer cells from P11 not only coexpressed *NKX2-1* and *TP63* but also showed high expression of *CHGB* and *UCHL1*, which are markers of neuroendocrine cells [41] (Additional file 1: Fig. S4a). Multiplex fluorescent IHC staining of TTF1 and UCHL1 on tumor tissues from P11 also verified the coexpression of these two markers in a high proportion of tumor cells (Additional file 1: Fig. S4c). Moreover, we confirmed that *NKX2-1* and *TP63* were coexpressed in the same tumor cells by RNA in situ hybridization (Fig. 2e; Additional file 3). In summary, we verified that mixed-lineage cancer cells are prevalent in many different patients with NSCLC.

### Mixed-lineage and single-lineage tumor cells in the same patient originate from common tumor ancestor cells

**Fig. 3:**
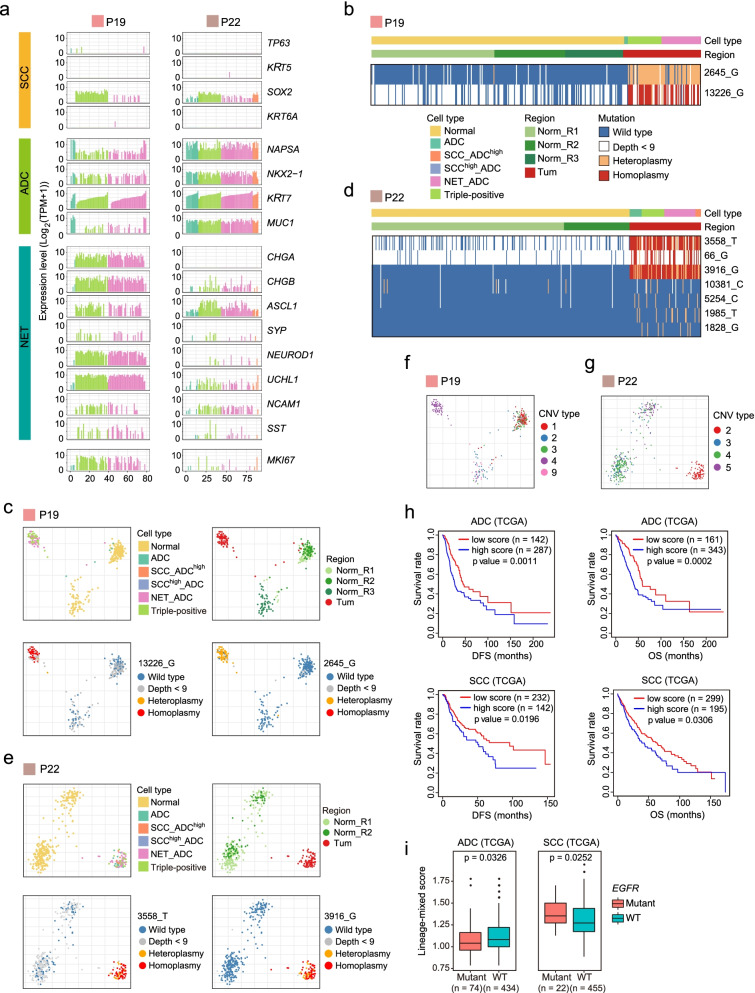
Mixed-lineage and single-lineage tumor cells in the same patient originate from common tumor ancestor cells. **a** Line plots showing the gene expression levels of clinical lineage-specific markers for single tumor cells from P19 and P22. **b** Heatmap showing the identified mitochondria mutations specific to tumor epithelial cells from P19. **c** PCA plot of single cells from P19, colored by redefined cell type, sample regions, and selected mitochondrial mutations. **d** Heatmap showing identified mitochondria mutations specific to tumor epithelial cells from P22. **e** PCA plot of single cells from P22, colored by cancer type, sample regions, and selected mitochondrial mutations. **f** PCA profiles of single cells from P19, colored by CNV clusters inferred by single-cell RNA-seq data. Only the cells with a corresponding CNV subtype with more than 10 cells were used for PCA analysis. **g** PCA profiles of single cells from P22, colored by CNV clusters inferred by single-cell RNA-seq data. Only the cells with a corresponding CNV subtype with more than 10 cells were used for PCA analysis. **h** Survival analysis of disease-free survival (DFS) and overall survival (OS) in ADC and SCC samples from TCGA. Mixed-lineage features were calculated based on the expression levels of the identified lineage marker genes for ADC, SCC, and NET. Samples with high scores and low scores represent high lineage-mixing features and low lineage-mixing features, respectively. **i** Mixed-lineage features evaluation for *EGFR* mutant samples and wild-type samples in ADC and SCC samples from TCGA, respectively. Mixed-lineage features were calculated based on the expression levels of the identified lineage marker genes for ADC, SCC, and NET. The higher the score indicates the higher lineage-mixed features

Patients P19 and P22 possessed tumors with two combined components. We found that for patient P19, who had C-SCLC, most cells highly expressed not only the NET markers *CHGA*, *CHGB*, *ASCL1*, and *NEUROD1* but also the ADC marker *NAPSA*. Only a small fraction of cancer cells solely expressed ADC markers. For patient P22 with MANEC, almost all cancer cells highly expressed *NAPSA*, and some of them coexpressed *ASCL1*. Therefore, unlike ADC patients P5, P10, P11, and P13, patients P19 and P22 mainly had NET and ADC mixed-lineage cancer cells (Fig. 3a). The mixed-lineage cancer cells showed mixed features of different subtypes of NSCLC, indicating that they were highly plastic.

To determine the relationship of mixed-lineage cancer cells and single-lineage cancer cells in the same patient, we investigated the cancer phylogenetic structure based on our scRNA-seq data using mitochondrial mutation-based lineage tracking analysis and single-cell copy number variation (CNV) analysis following previously described methods [33, 34, 42]. We identified two tumor-specific mutations, 2645_G and 13226_G, in patient P19 (Fig. 3b). According to the cancer type identification above, tumor cells from P19 were mainly composed of the NET_ADC and triple-positive subtypes. Interestingly, the NET_ADC and triple-positive cancer subtypes shared these two mutations, suggesting that these two types of mixed-lineage cancer cells had common tumor ancestors in patient P19 (Fig. 3c). In addition, we identified more mitochondrial mutation sites specific to tumor cells for patient P22, such as 3558_T, 66_G, 3916_G, 5254_C, and 1985_T (Fig. 3d). These mutations were shared by all four cancer subtypes: ADC, NET_ADC, SCC_ADC^high^, and triple-positive (Fig. 3d, e). Therefore, the mitochondrial mutation results indicated that the mixed-lineage cancer cells and ADC-based single-lineage cancer cells in the same patient had common tumor cell ancestors. Although we can not deduce the direction of the lineage changes, the most likely scenario is that in a NSCLC patient, a specific lineage of the normal epithelial cells was first transformed to single-lineage tumor cells during tumorigenesis, which further changed to mixed-lineage tumor cells. This does not exclude the possibility that at the late stage of tumorigenesis the mixed-lineage and single-lineage tumor cells can interchange easily with each other due to their plasticity and flexibility.

We next performed a single-cell CNV analysis based on the scRNA-seq data to further support the results of mitochondrial mutation analysis. We selected single-cell gene expression data from all normal epithelial cells as a control to calculate the CNVs of tumor epithelial cells. As demonstrated in Fig. 3f and Additional file 1: Fig. S5a, we found that two mixed subtypes, NET_ADC and triple-positive, in patient P19 had the same CNV patterns. To validate the accuracy of our single-cell CNVs, we assessed CNVs using the data generated from whole-genome sequencing (WGS) on bulk cells from the same patient and found that the results were consistent with these CNVs inferred by single-cell RNA-seq analysis (Additional file 1: Fig. S5b). For example, gain of chromosome 3 and chromosome 5 and loss of chromosome 4 in tumor cells were clearly evident in CNVs inferred by both scRNA-seq and bulk WGS. For patient P22, ADC cells and other mixed-lineage cancer cells also had the same CNV patterns (Fig. 3g and Additional file 1: Fig. S5c). As a result, we further confirmed that the mixed-lineage cancer cells and single-lineage cancer cells in the same patient had common tumor ancestors. Interestingly, we also found that a number of individual cells from adjacent normal tissues had copy number losses in chromosomes 4q and 8p (Additional file 1: Fig. S5a, c, d).

Intratumor heterogeneity contributes to clinical therapy failure and tumor progression [43]. Our scRNA-seq results highlighted the molecular heterogeneity and diversity in an individual tumor of NSCLC containing mixed-lineage cancer cells. These mixed cancer cells may have an aberrant cellular differentiation program or be associated with the transformation between the different subtypes. To analyze the relationship between mixed-lineage cancer cells and prognosis, we partitioned bulk RNA-seq samples of NSCLC from The Cancer Genome Atlas (TCGA) into two clusters based on the calculated lineage-mixing score. Patients with high lineage-mixing features (high score) were correlated with decreased survival, which indicates that a higher percentage of cells with mixed-lineage features in NSCLC predict poorer prognosis (Fig. 3h). Since data of TCGA were generated on bulk samples, it is possible that instead of expressing multi-cancer subtype markers in the same individual cell, marker genes of different cancer subtypes were sepatately expressed in different subpopulations of cancer cells in the tumor tissue. But the score can still reflect the general mixed trend of different cancer subtypes in the tumor tissues. To further investigate the potential connections between mixed-lineage cancer cells and *EGFR* mutation, we partitioned TCGA samples into two clusters based on *EGFR* mutation, *EGFR*^WT^ and *EGFR*^Mut^. We found that for ADC, the patients with *EGFR* mutation have lower mixed-lineage features than those with wild-type *EGFR*. In contrast, for SCC, the patients with *EGFR* mutation have higher mixed-lineage features than the patients with wild-type *EGFR* (Fig. 3i). Combined with the anticancer therapeutic effect of drugs against *EGFR* mutation, the ADC patients with mutated *EGFR* have better responses to epidermal growth factor receptor tyrosine kinase inhibitors (EGFR-TKIs) than the SCC patients. We speculate that the heterogeneity of mixed-lineage cancer cells may underlie variants in therapeutic responses to the EGFR-TKI target therapy between ADC and SCC patients.

### Transcriptome dynamics analysis reveals gene regulation changes during tumorigenesis

To determine the transcriptional signatures of tumor cells, DEGs were identified between all normal epithelial cells and tumor epithelial cells. Eighty downregulated genes and 225 upregulated genes (log_2_fold change (tumor versus normal) > 2) were identified in tumor epithelial cells. Then, we constructed a pseudotime trajectory to uncover the transcriptome changes during tumorigenesis from the normal epithelium (left) to carcinoma (right) (Additional file 1: Fig. S6a). The genes whose expression levels changed along the trajectory were grouped into 4 distinct clusters based on the dynamic expression patterns (Additional file 2: Table S2). To better understand the biological significance of each cluster of genes, we performed a Gene Ontology (GO) analysis (Additional file 1: Fig. S6b). Genes in cluster 1 were dramatically downregulated in cancer epithelial cells. GO analysis showed that these genes were mainly involved in the regulation of defense (response to bacterium and antimicrobial humoral response), homeostasis, and activation of innate immunity by increasing cell chemotaxis and cytokine levels. Genes in clusters 2 and 3 were widely upregulated in tumor epithelial cells. Genes in cluster 2 were mainly enriched in the immune response to the virus and interferon signaling pathway, and genes in cluster 3 were enriched for the following GO terms: extracellular matrix organization, response to toxic substances, and P53 signaling pathway. Genes in cluster 4, which are mainly highly expressed in late-stage tumors, were strongly enriched in cell proliferation-related terms, including cell division, cell cycle, and mitosis, thus indicating why late-stage tumor cells are more likely to proliferate and metastasize.

### *AKR1B1* is necessary for tumor cell growth

**Fig. 4:**
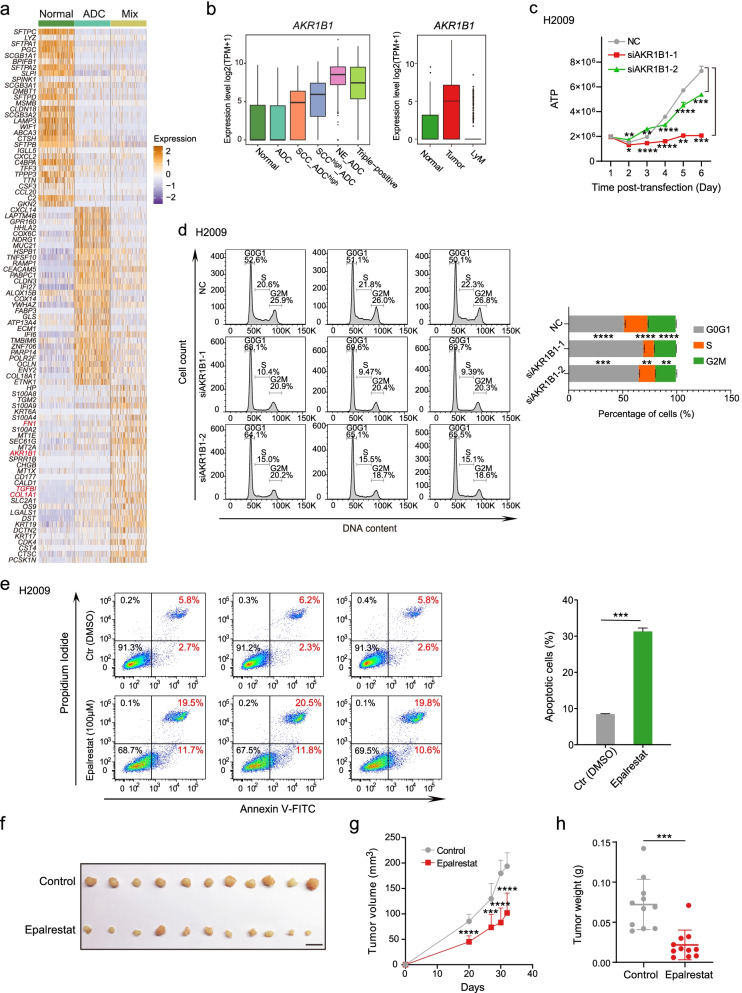
AKR1B1 is essential for the proliferation of lung tumor cells. **a** Heatmap showing differentially expressed genes among normal epithelial cells, ADC-based single-lineage tumor cells, and combined four mixed-lineage tumor cells. **b** Box plot in the left shows the single-cell gene expression level of *AKR1B1* in normal epithelial cells and five cancer cell subtypes. Box plot in right shows the single-cell gene expression levels of *AKR1B1* in epithelial cells from normal tissues, tumor tissues, and LyM tissues. LyM represents lymph node metastasis. **c** Proliferation analysis of H2009 cells after *AKR1B1* was knockdown with two different siRNAs. Compared with non-targeting control (NC), siAKR1B1-1 and siAKR1B1-2 significantly reduced the proliferation of H2009 cells. *p < 0.05; **p < 0.01; ***p < 0.001; ****p < 0.0001. *p* values were determined by *t*-test. **d** Cell cycle analysis after H2009 cells were transfected with two different siRNAs of *AKR1B1* for 48 h. Compared with NC, both siAKR1B1-1 and siAKR1B1-2 significantly decreased the cell fractions of S and G2/M phases of the cell cycle. *p < 0.05; **p < 0.01; ***p < 0.001; ****p < 0.0001. *p* values were determined by *t*-test. **e** Cell apoptosis analysis after H2009 cells were treated with DMSO or 100 μM epalrestat for 48 h. Epalrestat treatment significantly promoted the apoptosis of H2009 cells. ****p < 0.001. *p* values were determined by *t*-test. **f** Photograph of tumors treated with sterile water or epalrestat 36 days after injection. These 11 tumors in the control group were derived from 7 mice, and these tumors in the treatment group were derived from 6 mice. Scale bar, 10 mm. **g** Quantitation of tumor volumes. The tumor volumes were calculated by the following formula: volume = length × (width)^2^ × 0.5. The maximum and minimum detected in each time point were removed. ***p < 0.001; ****p < 0.0001. *p* values were determined by *t*-test. **h** Quantitation of tumor weight from tumors in **f**. Epalrestat treatment significantly inhibited tumor growth. ***p < 0.001. *p* values were determined by *t*-test

To further investigate the molecular signatures that were involved in mixed-lineage features of tumor cells, we identified the DEGs among normal epithelial cells, ADC-based single-lineage tumor cells, and combined mixed-lineage tumor cells (Fig. 4a; Additional file 2: Table S3). We found that mesenchymal-related markers such as *FN1*, *TGFBI*, and *COL1A1* were enriched in mixed-lineage tumor cells. It is known that tumor transformation may occur via EMT, during which process epithelial cells acquire mesenchymal-related features [44, 45]. In addition, epithelial cell differentiation regulation-related genes, such as *AKR1B1*, *SPRR1B*, and keratin genes *KRT6A*, *KRT19*, and *KRT17* were also highly expressed in mixed-lineage tumor cells*.* Specially *AKR1B1* displayed high expression in all four mixed-lineage tumor subtypes in tumor tissues (Fig. 4b). AKR1B1 is involved in the glucose transforming polyol pathway and has been reported to have the capacity to facilitate breast cancer tumorigenesis and metastasis via EMT process [46]. In addition, a study also showed that the expression of AKR1B1 was strongly correlated with EMT in lung cancer and colon cancer, during which process that tumor cells could obtain properties of cancer stem cells manifesting diverse plasticity [47]. Therefore, we speculated that AKR1B1 may be one of the master regulators of mixed-lineage tumor cells' plasticity. To further understand the underlying molecular mechanisms of the tumorigenesis of mixed-lineage tumor cells, we focused on functional analysis of AKR1B1. First, we performed siRNA knockdown experiments with two different siRNAs of *AKR1B1* in the H2009 cell line (Additional file 1: Fig. S7a). Knockdown of *AKR1B1* can significantly decrease the proliferation of H2009 cells compared with the non-targeting siRNA control (NC) (Fig. 4c). Moreover, knockdown of *AKR1B1* strongly reduced the proportion of cells in the S phase and G2/M phase of the cell cycle (Fig. 4d).

Next, we treated H2009 cells with 100 μM epalrestat, a specific inhibitor of AKR1B1 that has been approved for the treatment of diabetes complications [48]. As demonstrated in Additional file 1: Fig. S7b, we observed that the cell numbers in the epalrestat treatment group also decreased compared to those in the control group (treated with DMSO). To further exclude the potential side effects caused by the high concentration of epalrestat and to test the drug specificity, the colon cancer cell line SW480, which essentially does not express *AKR1B1* according to the Cancer Cell Line Encyclopedia (CCLE) RNA-seq data (Additional file 1: Fig. S7c), was treated with the same concentration of epalrestat. After H2009 cells and SW480 cells were treated with 100 μM epalrestat in parallel for 48 h, the percentage of apoptotic cells was significantly higher in H2009 cells but not in SW480 cells, suggesting that epalrestat can specifically promote apoptosis by inhibiting AKR1B1 (Fig. 4e; Additional file 1: Fig. S7d). There is a minor possibility that the different tissue origins of H2009 cells and SW480 cells may also cause the differences in response to epalrestat between these two cancer cell lines. In addition, we performed bulk RNA-seq to analyze the gene expression in epalrestat treated cells and control (DMSO only) cells. We identified 1540 upregulated genes and 1737 downregulated genes (fold change (epalrestat versus DMSO) > 1.5, P value < 0.01) in epalrestat treated H2009 cells (Additional file 4: Table S4). As epalrestat is a non-competitive inhibitor of aldolase reductase, we found that when treating H2009 cells with epalrestat to inhibit the activity of aldolase reductase, the expression of *AKR1B1* was slightly upregulated potentially though a negative feedback regulation mechanism. AKR1B1 could be involved in different metabolic and physiological processes and participate in a complex network of signaling pathways, such as inflammation, cell cycle, epithelial to mesenchymal transition, and mTOR pathway [49]. By analyzing the differentially expressed genes, we found that downregulated genes in epalrestat treated cells were mainly enriched in cell-cell adhesion, positive regulation of cell migration, negative regulation of cell differentiation, cell cycle, mitotic, prostaglandin biosynthetic process, and related metabolic processes, consistent with the proposed function of AKR1B1 (Additional file 1: Fig. S7e). Upregulated genes were mainly enriched in the cellular response to extracellular stimulus, response to nutrient levels, and cellular response to glucose starvation-related biological processes. In addition, positive regulation of the apoptotic process was also enriched in these upregulated genes, which was consistent with the observation that epalrestat treatment group samples had a higher proportion of apoptotic cells (Additional file 1: Fig. S7e). To further examine the effect of epalrestat on tumorigenesis in vivo, we injected H2009 cells into NOD-SCID mice. Then, the mice were intragastrically administered epalrestat daily. One month later, the tumors were collected for further analysis. Compared to control mice, epalrestat-treated mice exhibited significantly reduced tumor cell growth in vivo (Fig. 4f–h). In summary, these data demonstrated that *AKR1B1* could play important roles in the tumorigenesis of lung cancer. However, as we did not detect the effectiveness of epalrestat on AKR1B1 target for tumor cells of mice, further evaluations on the potential off-target effectiveness of epalrestat in vivo should be carried out in the future.

To further understand the transcriptomic regulation of *AKR1B1*, we performed bulk RNA-seq to analyze the gene expression in H2009 cells with *AKR1B1* knockdown. We identified 143 upregulated genes and 473 downregulated genes (fold change (siAKR1B1 versus NC) > 2, *p* value < 10^−15^) in H2009 cells with *AKR1B1* knockdown (Additional file 1: Fig. S7f; Additional file 5: Table S5). Downregulated genes were mainly enriched in metabolism-related pathways, consistent with the known function of *AKR1B1*. In addition, cancer-related pathways, such as the p53 signaling pathway, the HIF-1 signaling pathway, focal adhesion, and cell cycle DNA replication, were also enriched in these downregulated genes, which is in line with the observation that knockdown samples had lower levels of proliferation characteristics (Additional file 1: Fig. S7f). Upregulated genes were mainly enriched in biosynthetic processes and negative regulators of cell proliferation (Additional file 1: Fig. S7g). To further explore the inhibition specificity of epalrestat toward AKR1B1, we compared these differentially expressed genes (DEGs) (fold change (epalrestat versus DMSO) > 1.5, *p* value < 0.01) of epalrestat-treated cells with those DEGs (fold change (siAKR1B1 versus NC) > 1.5, *p* value < 0.01) of siAKR1B1-treated cells. We identified 1179 upregulated genes and 1684 downregulated genes in siAKR1B1 treated cells. When we merged the DEGs of these two datasets, we found 84 overlapped upregulated genes and 170 overlapped downregulated genes (Additional file 6: Table S6). The overlapped downregulated genes were involved in many pathways, such as interferon alpha/beta signaling, response to decreased oxygen levels, positive regulation of cell migration, metabolism of carbohydrates, glucose metabolism, regulation of protein serine/threonine kinase activity, cell cycle, and cell population proliferation, which were correlated with the mentioned functions of AKR1B1 (Additional file 7: Table S7). The overlapped upregulated genes were mainly enriched in the metabolism and transport process, such as cellular amide metabolic process, tetrapyrrole metabolic process, long-chain fatty acid transport, and monocarboxylic acid transport. In addition, other GO terms such as cellular component morphogenesis, epithelial cell differentiation, and negative regulation of cell population proliferation were also enriched in these upregulated genes (Additional file 7: Table S7). Although analysis of common DEGs of epalrestat-treated cells and siAKR1B1-treated cells revealed that these overlapped genes enriched GO terms were consistent with the functions of AKR1B1, significant differences were also observed between these two datasets. Considering that our RNA sequencing results of siAKR1B1-treated cells were more reliable with 90% knockdown efficiency of *AKR1B1*, we speculated that epalrestat may have some off-target effects in the H2009 cell line. It has been reported that epalrestat also has a binding affinity toward AKR1B10, a member of aldo–keto reductase superfamily, and could suppress the enzymatic activity of AKR1B10. Therefore, we speculated that epalrestat may have some off-target effects in the H2009 cell line by targeting other members of aldo–keto reductase superfamily, such as AKR1B10, but the in-depth mechanism of AKR1B1 inhibition mediated by epalrestat in lung cancer need to be further investigated in the future. Collectively, our functional analysis of *AKR1B1* showed that *AKR1B1* was necessary for tumor growth and elucidated its transcriptomic regulation in NSCLC.

## Discussion

NSCLC is a complex disease and can be categorized into several cancer subtypes. The accurate diagnosis of the NSCLC subtypes plays a pivotal role in clinical treatment. The 2011 WHO classification for lung cancer elucidated the need for IHC in classification and diagnosis [50]. The use of IHC has improved the accuracy of classification, particularly for poorly differentiated tumors that are difficult to judge cancer subtypes from the histopathological characteristics. However, it is still challenging and tricky when selecting different antibodies or antibody batches. scRNA-seq technology as a powerful tool can provide the whole transcriptome information at single-cell resolution, which makes it feasible to dissect the heterogeneity of complex tumors. Here, we performed a high-precision single-cell transcriptome analysis of cells from primary lung tumor tissues and matched normal tissues of NSCLC patients. By focusing on the transcriptome of cancer cells, we comprehensively analyzed the molecular characteristics of NSCLC. For the first time, we identified a significant proportion of mixed-lineage cancer cells co-expressing different cancer subtype lineage markers in the same individual cells in many NSCLC patients at the single-cell whole-transcriptome level. We further validated the finding by both multiplex fluorescent IHC staining and RNA in situ hybridization. Although three recent papers reported three unusual cases of NSCLC with co-expression of TTF1 (NKX2-1) and p40 (TP63) in the same cells by IHC staining [51–54], they failed to observe the molecular heterogeneity present in an individual tumor. Our single-cell whole-transcriptional analysis of NSCLC is a powerful tool that provides molecular heterogeneity and cellular diversity in individual tumors and gives great promise in a more accurate classification of NSCLC.

The major subtypes of lung cancer—ADC, SCC, and NETs—are considered different diseases originating from distinct lineages of epithelial cells and thus have very different clinical treatment strategies. The cancer phylogenetic structure analysis using mitochondrial mutation-based lineage tracking analysis and single-cell CNV analysis based on our scRNA-seq data revealed that the mixed-lineage tumor cells and single-lineage tumor cells in the same patient actually originated from common tumor ancestors. This also indicates that, regardless of which normal epithelial cell the tumor cells originated, these mixed-lineage tumor cells are highly plastic to acquire double- or even triple-lineage features (ADC, SCC, NET) during tumorigenesis, and this multi-lineage plasticity of the cancer cells is probably connected to their prevalent drug resistance in NSCLC patients. Upon linking the cellular plasticity of mixed-lineage tumor cells with the mixed-lineage features of different subtypes of NSCLC, we hypothesized that these mixed-lineage tumor cells contribute to tumor transformation, drug insensitivity, and therapeutic resistance in NSCLC. The survival analysis indicated that the patients with higher mixed-lineage features of different NSCLC subtypes had decreased survival time. By combining the transcriptomic analysis and molecular characterization, these mixed-lineage cancer cells are worth further study and investigation.

To uncover the molecular regulation of NSCLC, we constructed a pseudotime trajectory from the normal epithelial cells to the cancer cells. We identified a tumor-specific gene set and revealed the gene expression dynamics during tumorigenesis. Furthermore, we identified gene signatures specific to mixed-lineage tumor cells including *AKR1B1*. We found that *AKR1B1* was significantly upregulated in all mixed-lineage tumor cells of the majority of patients we analyzed. AKR1B1 is involved in the polyol pathway of glucose metabolism and has been reported to have the capacity to facilitate breast cancer, lung cancer, and colon cancer tumorigenesis via EMT [46, 47]. Our functional analysis of *AKR1B1* showed that it can promote lung tumor proliferation and growth both in vitro and in vivo and play an important role in tumorigenesis, such as the regulation of the p53 signaling pathway and cell cycle. This suggests that *AKR1B1* can serve as a candidate target for tumor therapy of NSCLC patients with mixed-lineage features. Collectively, our study provides novel insights into NSCLC and offers clues for the more refined classification, diagnosis, and treatment of its various subtypes.

## Conclusions

In conclusion, we utilized high-precision single-cell RNA-seq analysis to dissect the molecular heterogeneity of cancer cells of human NSCLC. We identified a subpopulation of mixed-lineage tumor cells and found that these mixed-lineage tumor features were associated with a poorer prognosis. Furthermore, gene signatures specific to mixed-lineage tumor cells were identified including AKR1B1. Gene knockdown experiment verified that AKR1B1 is necessary for tumor growth, suggesting that it can serve as a candidate target for tumor therapy of NSCLC patients with mixed-lineage features. These results provide a high-resolution overview of tumor cells of human NSCLC and highlight a novel mixed-lineage tumor subpopulation that may contribute to tumor progression and tumor transdifferentiation between different subtypes of NSCLC and offer clues for potential therapeutic strategies for these patients with mixed-lineage features.

## Supplementary Information

**Additional file 1: Fig. S1.** Hematoxylin eosin (H&E) staining of samples in this study. **Fig. S2.** Patient information and quality control of single-cell RNA-seq data. **Fig. S3.** Transcriptome analysis of different cancer subtypes. **Fig. S4.** Mixed-lineage cancer cells identification at single-cell transcriptome level and at protein level. **Fig. S5.** Copy number variation analysis. **Fig. S6.** Pseudotime analysis revealed transcriptome dynamics during tumorigenesis. **Fig. S7.** Knockdown and inhibition of AKR1B1 decreased tumor cell growth.

**Additional file 2: Table S1.** Cell lineage markers identified for ADC, SCC and NET cancer subtypes. **Table S2.** Differentially expressed genes during tumorigenesis. **Table S3.** Differentially expressed genes among normal epithelial cells, ADC-based single-lineage tumor cells and combined mixed-lineage tumor cells.

**Additional file 3:** **Movie S1.** RNA in situ hybridization of cells from ADC tissues with *NKX2-1* and *TP63*. Green, *NKX2-1*; red, *TP63*.

**Additional file 4:** **Table S4.** Differentially expressed genes between epalrestat treated samples and DMSO treated samples.

**Additional file 5:** **Table S5.** Differentially expressed genes between siAKR1B1 and NC samples.

**Additional file 6: Table S6.** Overlapped differentially expressed genes between epalrestat treated samples and siAKR1B1 treated samples.

**Additional file 7:** **Table S7.** GO terms enriched in overlapped upregulated genes and GO terms enriched in overlapped downregulated genes of epalrestat treated samples and siAKR1B1 treated samples.

## Data Availability

The transcriptome raw data and the whole-genome sequencing raw data generated in this study have been deposited in the Genome Sequence Archive (GSA) with accession number HRA000270 (https://ngdc.cncb.ac.cn/gsa-human/browse/HRA000270) [55]. All other relevant data are available on request from the authors. The code used for the graphic presentation is available on GitHub (https://github.com/WRui/) [56].
