# Supplementary material for: Molecular profiling of human non-small cell lung cancer by single-cell RNA-seq
Source: Genome Med. 2022 Aug 13;14:87. doi: 10.1186/s13073-022-01089-9 (PMC9375433; doi:10.1186/s13073-022-01089-9)
Supplement: Supplementary file 1 — Additional file 1: Fig. S1. Hematoxylin eosin (H&E) staining of samples in this study. Fig. S2. Patient information and quality control of single-cell RNA-seq data. Fig. S3. Transcriptome analysis of different cancer subtypes. Fig. S4. Mixed-lineage cancer cells identification at single-cell transcriptome level and at protein level. Fig. S5. Copy number variation analysis. Fig. S6. Pseudotime analysis revealed transcriptome dynamics during tumorigenesis. Fig. S7. Knockdown and inhibition of AKR1B1 decreased tumor cell growth. [file 13073_2022_1089_MOESM1_ESM.docx]

**Supplementary Information**

**Additional file 1:** Supplementary Materials for: Molecular profiling of human non-small cell lung cancer by single-cell RNA-seq. This file includes Supplementary Figures: Figs. S1-S7

**Fig. S1** Hematoxylin eosin (H&E) staining of samples in this study.

**Fig. S2** Patient information and quality control of single-cell RNA-seq data.

**Fig. S3** Transcriptome analysis of different cancer subtypes.

**Fig. S4** Mixed-lineage cancer cells identification at single-cell transcriptome level and at protein level.

**Fig. S5** Copy number variation analysis.

**Fig. S6** Pseudotime analysis revealed transcriptome dynamics during tumorigenesis.

**Fig. S7** Knockdown and inhibition of AKR1B1 decreased tumor growth.

**
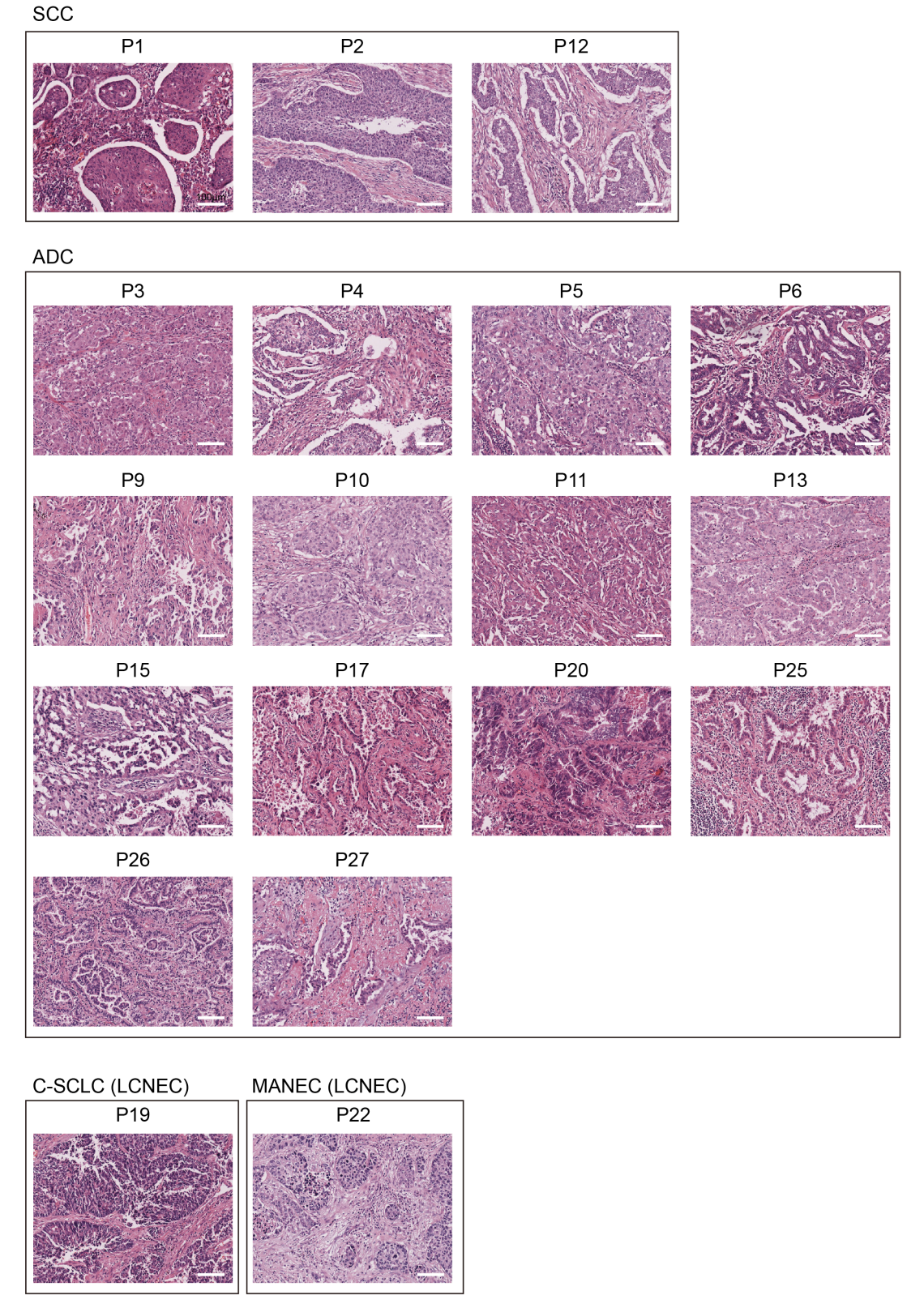
**

**Fig. S1** Hematoxylin eosin (H&E) staining of samples in this study. In this study, we analyzed three lung squamous cell carcinoma (SCC) patients, fourteen lung adenocarcinoma (ADC) patients and two mixed-component patients including C-SCLC patient P19 and MANEC patient P22. The cancer subtypes were determined by combination of light microscopy using hematoxylin and eosin-stained slides and IHC for classical markers. Scale bar, 100 μm.

**
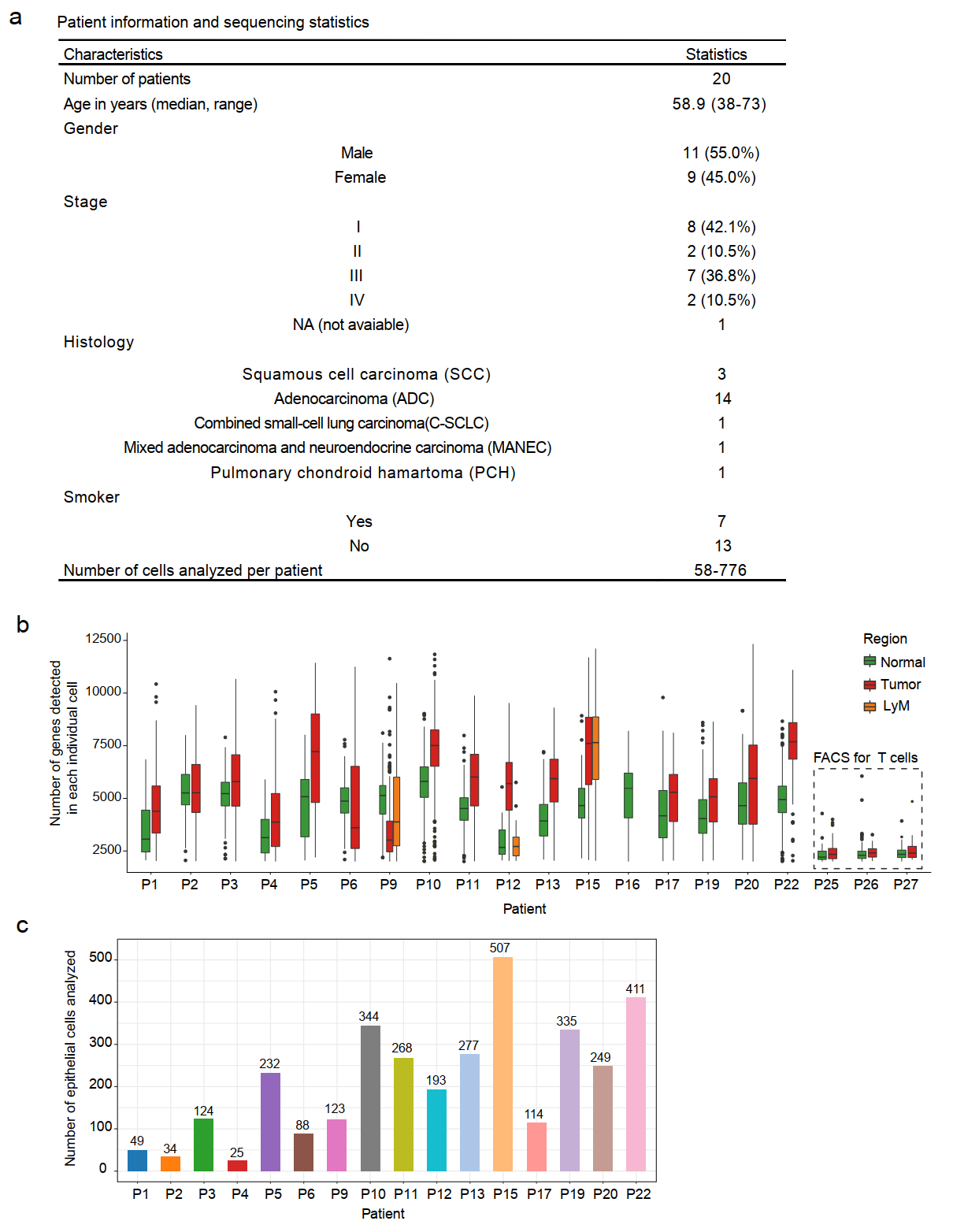
**

**Fig. S2** Patient information and quality control of single-cell RNA-seq data. **a** Clinical information of the patients analyzed in this study and the numbers of cells passed quality control. **b** Box plot displayed gene numbers detected of single cells from different tissues across each patient after filtering for at least 2000 genes detected in each individual cell. With the exception of single cells from patients P25, P26 and P27 for whom CD3^+^ T cells were sorted by fluorescence-activated cell sorting (FACS), for the rest of the patients, single cells were randomly picked without preselection for specific cell types. LyM represents lymph node metastasis. **c** Statistics of cell numbers for epithelial cells contributed to each patient.


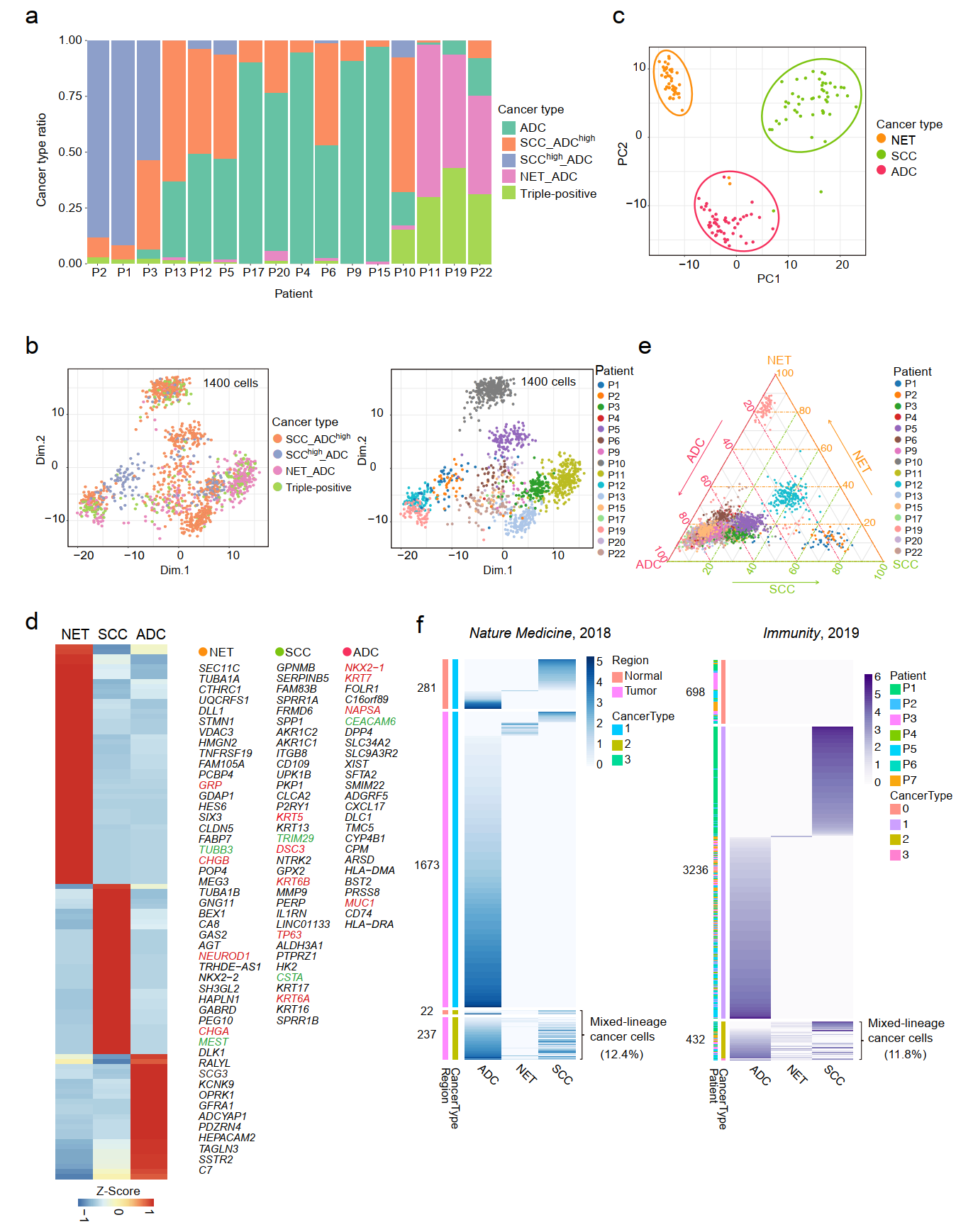


**Fig. S3** Transcriptome analysis of different cancer subtypes. **a** Barplot showing identified cancer type ratios in tumor tissues across every patient. **b** PCA plot for 1400 mixed-lineage tumor cells. Cells are colored by identified cancer types and patients, respectively. **c** PCA plot showing top 50 cells for each lineage highly expressing corresponding lineages markers were well separated from each other lineage. **d** Heatmap showing cancer subtype markers of NET, ADC, and SCC identified from DEG analysis from cells in **c**. The known cancer lineage markers were colored by red, and the new identified candidate markers for each lineage were colored by green. **e** The ggtern plot for all tumor epithelial cells. The colors indicated cells from different patients. **f** Heatmap showing expression score of lineage-specific marker genes in each epithelial cells from two previous published scRNA-seq datasets.

**
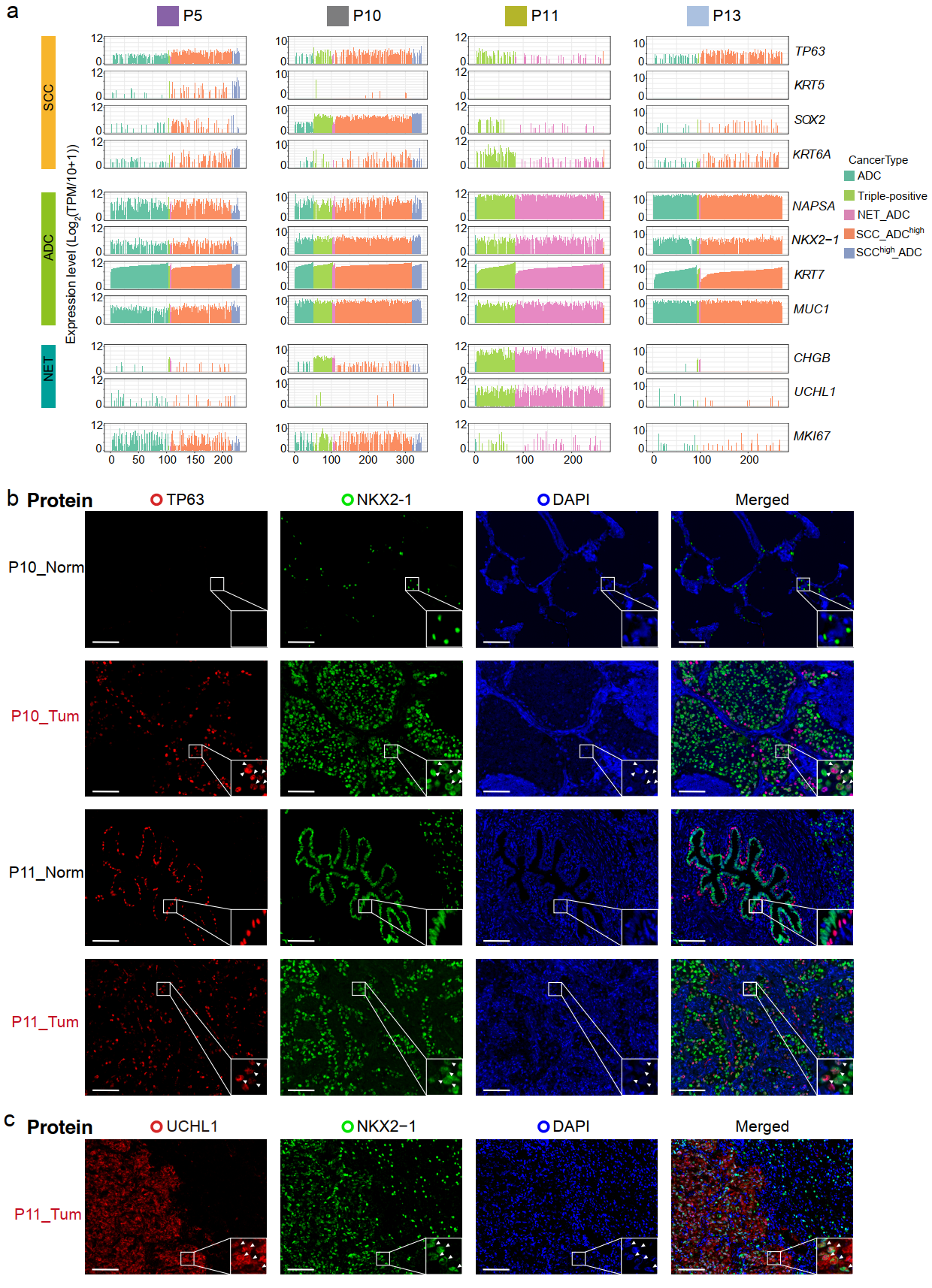
**

**Fig. S4** Mixed-lineage cancer cells identification at single-cell transcriptome level and at protein level. **a** Line plots showing gene expression levels of three typical lineage markers for single cell of tumor tissues from patients P5, P10, P11, and P13. The color indicated redefined cancer types. **b** Multiplex fluorescent IHC staining of cells from patients P10 and P11 with p63 (TP63) and TTF1 (NKX2-1). Arrows indicated the double positive cells. Scale bar, 100 μm. **c** Multiplex fluorescent IHC staining of cells from patient P11 with UCHL1 and TTF1 (NKX2-1). Arrows indicated the double positive cells. Scale bar, 100 μm.


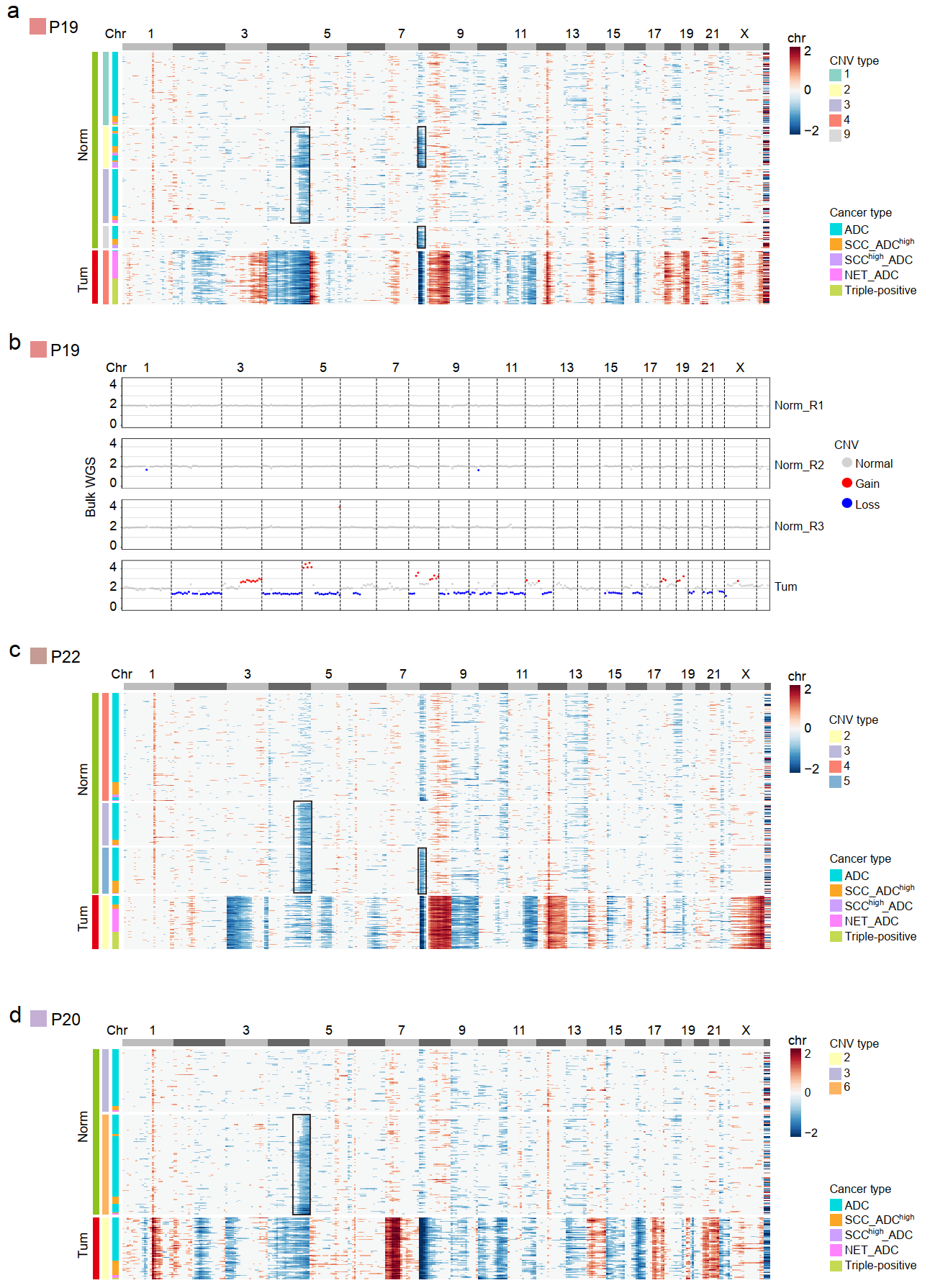


**Fig. S5** Copy number variation analysis. **a** Heatmap showing CNVs of single cells from patient P19. CNVs were inferred based on single cell average gene expressions. Only CNV types with more than 10 cells were kept. Red, gain; blue, loss. **b** CNVs confirmed by bulk whole-genome sequencing data. The samples include cells from tumor regions and three normal regions. **c**, **d** Heatmaps showing CNVs of individual cells from patients P20 and P22, respectively. CNVs were inferred based on single cell average gene expressions. Red, gain; blue, loss. Only CNV types with more than 10 cells were kept.


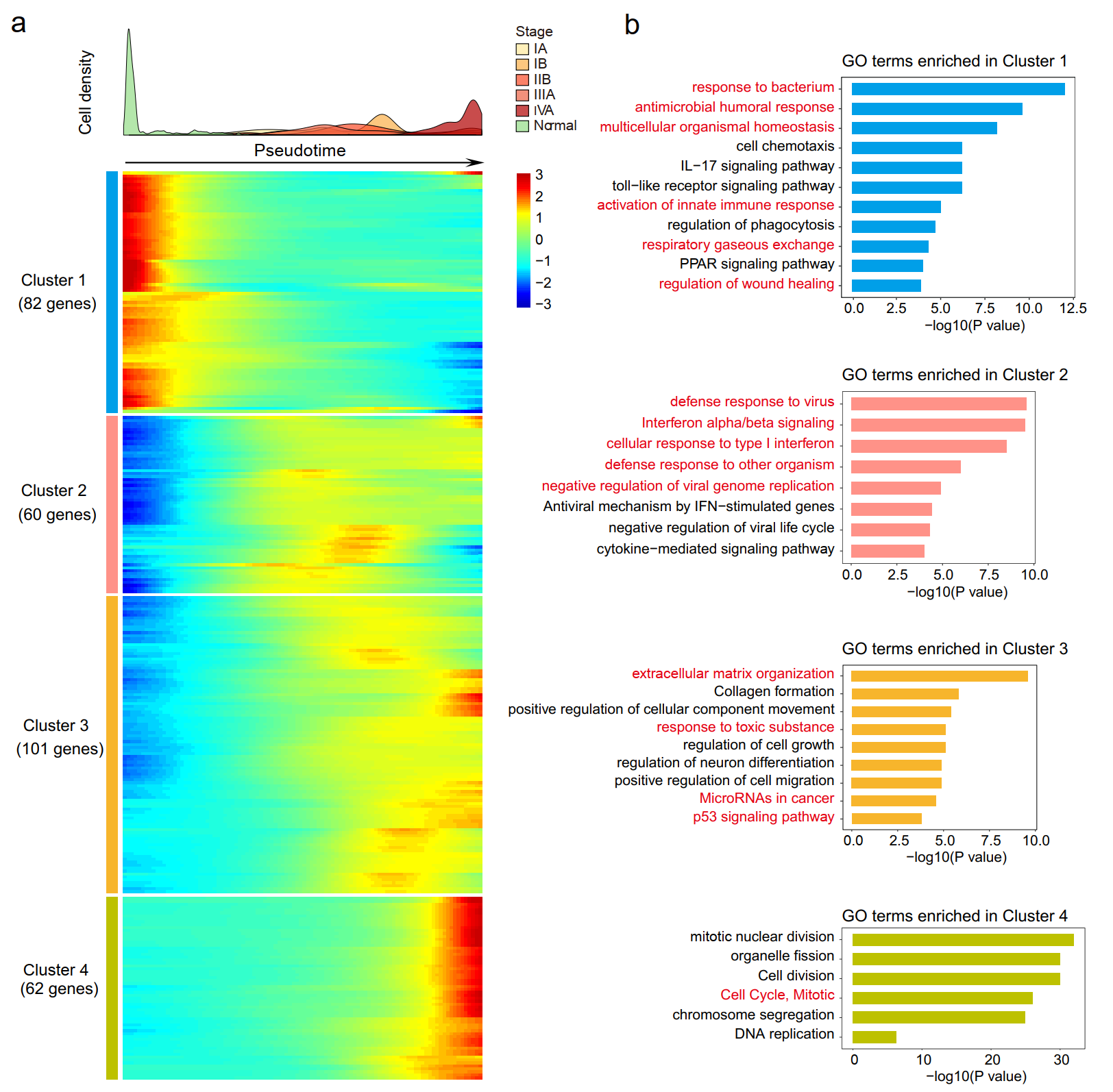


**Fig. S6** Pseudotime analysis revealed transcriptome dynamics during tumorigenesis. **a** Heatmap showing normalized gene expression dynamics along the pseudotime. Totally 305 identified DEGs between normal epithelial cells and tumor cells were grouped into four clusters based on their gene expression patterns along the constructed pseudotime. **b** Gene Ontology (GO) enrichment analysis of genes from four clusters grouped in **a**.

**
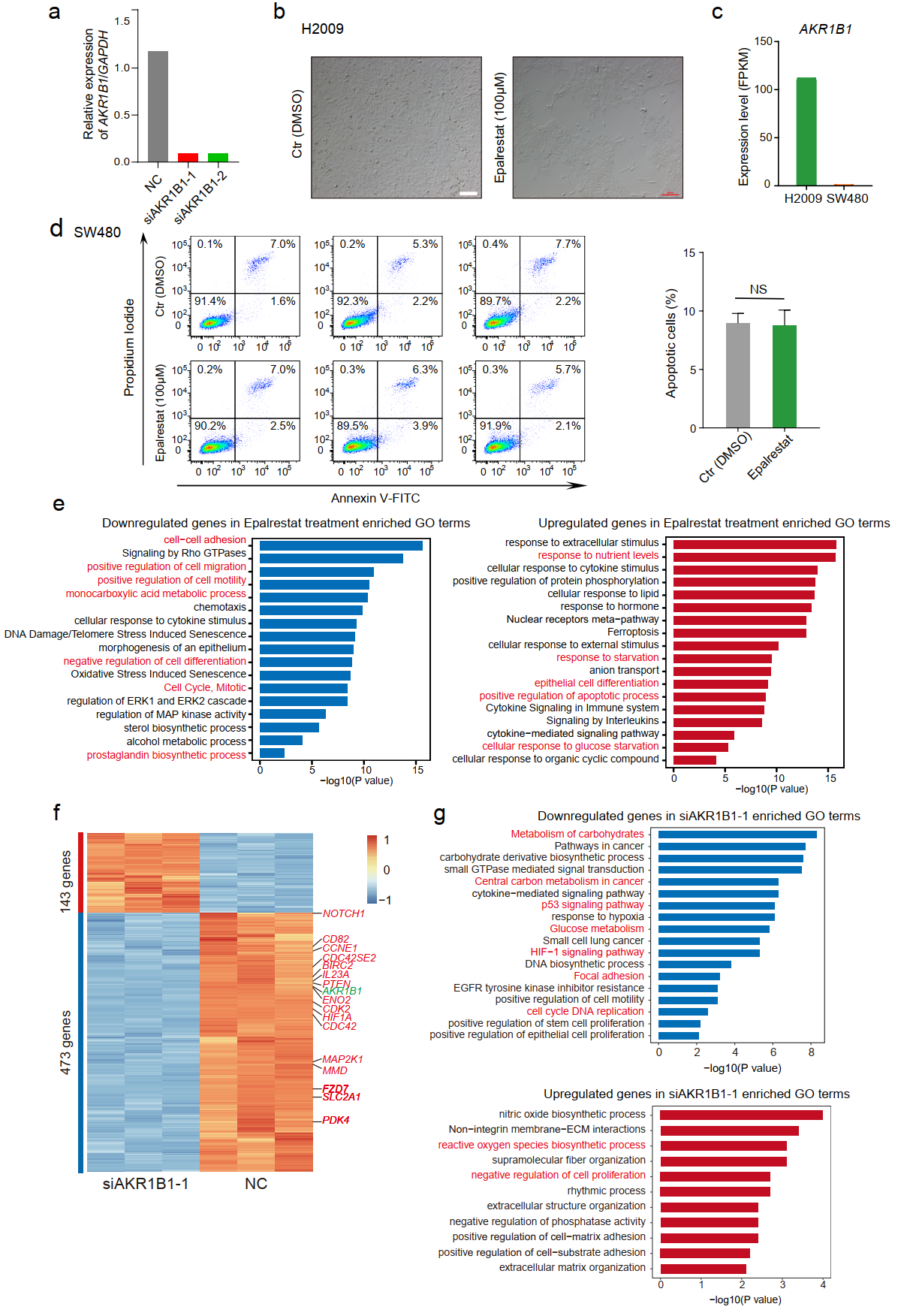
 Fig. S7** Knockdown and inhibition of AKR1B1 decreased tumor cell growth. **a** knockdown efficiency of two different siRNAs of AKR1B1 in H2009 cell line. **b** H2009 cells were treated with DMSO or 100 μM epalrestat for 48h. Photograph showing epalrestat treatment inhibited cell proliferation. Scale bar, 200 μm. **c** Expression levels of AKR1B1 in H2009 cells and SW480 cells obtained from CCLE database. **d** Cell apoptosis analysis after that SW480 cells were treated with DMSO or 100 μM epalrestat for 48h. The percentages of apoptotic cells between treatment with DMSO and epalrestat were not significant different (NS, not significant, *p* > 0.05).  *P* values were determined by *t*-test. **e** Selected GO terms enriched in downregulated genes and upregulated genes in epalrestat treatment group samples, respectively. **F** Heatmap showing differentially expressed genes (Fold change > 2, *p*-value < 10^-15^) between siAKR1B1 samples and NC samples by bulk RNA-seq. The orange and blue colors indicated upregulated genes and downregulated genes, respectively. **g** Selected GO terms enriched in downregulated genes and upregulated genes in siAKR1B1 samples, respectively.
